# A Versatile Capacitive Sensing Platform for the Assessment of the Composition in Gas Mixtures

**DOI:** 10.3390/mi11020116

**Published:** 2020-01-21

**Authors:** Jörgen Sweelssen, Huib Blokland, Timo Rajamäki, Risto Sarjonen, Arjen Boersma

**Affiliations:** 1 TNO, HTC25, 5656AE Eindhoven, The Netherlands; jorgen.sweelssen@tno.nl (J.S.); huib.blokland@tno.nl (H.B.); 2 National Metrology Institute VTT MIKES, Tekniikantie 1, FI-02150 Espoo, Finland; Timo.rajamaki@vtt.fi (T.R.); Risto.sarjonen@vtt.fi (R.S.)

**Keywords:** energy transition, gas composition sensor, capacitive sensor array, interdigitated electrodes, responsive coatings, tunable filter infrared spectrometer, LNG, biogas

## Abstract

The energy market is facing a major transition, in which natural gas and renewable gasses will play an important role. However, changing gas sources and compositions will force the gas transporters, gas engine manufacturers, and gas grid operators to monitor the gas quality in a more intensive way. This leads to the need for lower cost, smaller, and easy to install gas quality sensors. A new approach is proposed in this study that is based on the chemical interactions of the various gas components and responsive layers applied to an array of capacitive interdigitated electrodes. For Liquid Natural Gas (LNG), containing a relative high concentration of higher hydrocarbons, an array of ten capacitive chips is proposed, that is sufficient to calculate the full composition, and can be used to calculate energy parameters, such as Wobbe Index, Calorific Value, and Methane Number. A first prototype was realized that was small enough to be inserted in low and medium pressure gas pipes and LNG engine fuel lines. Adding the pressure and temperature data to the chip readings enables the determination of the concentrations of the various alkanes, hydrogen, nitrogen, and carbon dioxide, including small fluctuations in water vapor pressure. The sensitivity and selectivity of the new sensor is compared to a compact analyzer employing tunable filter infrared spectrometry.

## 1. Introduction

The Netherlands and other European regions are facing major changes during the coming decades in the production and use of natural gas for household heating and industrial processes. Both economic and political changes induce an accelerated reduction of the use of the nationally produced natural gas, and require a shift towards Liquid Natural Gas (LNG) from different sources all over the world and sustainable solutions such as biogas and hydrogen. All of these gasses have a deviating composition compared to the traditional sources. This requires a more intense monitoring of the composition along the gas grid and at end-user applications like LNG engines. The currently available gas quality measuring systems (e.g., Gas chromatograph, Wobbe Index analyzer, etc.) cannot fulfill the need for a cost-effective inline measuring method. Furthermore, the number of biogas feeds into an existing gas grid will increase significantly during the coming years. This not only asks for a clear monitoring of the distribution of this gas along the grid, but also an enhanced monitoring of the gas feed quality. Biogas may suffer from larger fluctuations in composition and accompanying contaminations. These should be recognized before entering a gas grid. Currently, gas chromatographs are used for these ‘gate keeper’ activities, but lower cost solutions may be required in order to facilitate starting biogas producers. In addition, LNG will play an increasingly important role as a transport fuel in LNG and dual-fuel engines. Monitoring gas compositions and the associated Methane Number (MN) will be critical for the optimal functioning of these engines when using LNG from different sources [1,2,3].

Many gas sensors have been presented for the detection of low concentrations of volatile organic compounds (e.g., [4]), among which also gas sensor arrays for e.g., food monitoring [5,6]. However, many of these solutions are based on detection principles that are not possible or allowed in calorific gas detection. Since there is no oxygen present in natural gas, the use of Metal Oxide or catalytic sensors is limited, because oxygen is required for regeneration. Furthermore, other detection principles, such as ionization [7] or heated sensing elements will not be allowed, because of safety reasons. 

Several sensor solutions have been proposed for the assessment of the composition and quality of natural gas, of which the majority is based on large laboratory equipment, such as Raman spectroscopy [8,9,10], gas chromatography [11], and infrared spectroscopy [12,13]. These laboratory analytical techniques perform very well in general with respect to selectivity and sensitivity, but are too costly for ubiquitous gas quality monitoring in gas grids or LNG engines. As a lower cost and smaller alternative, some new developments have been presented in the field of miniaturized MEMS based natural gas sensors [14,15,16], based on the optical interaction of infrared light and chemical species present in the gas. One of the major difficulties in infrared based detection of the components in natural gas, is the high chemical similarity of the hydrocarbon species. For this reason, an alternative gas sensor was developed that enables the differentiation of the various hydrocarbons in a natural gas mixtures based on the combination of chemical interaction, critical temperature, boiling point and size. This technology is based on the absorption of gas molecules in porous absorbing coatings applied to a capacitive sensing platform [17,18]. Some of the results, with respect to selectivity and sensitivity will be compared to a MEMS benchmark technology based on tunable filter infrared spectroscopy [19,20]. This paper will compare the performance of the two sensor technologies. 

## 2. Capacitive Gas Sensor Array Technology

### 2.1. Capacitive Interdigitated Electrodes

**Figure 1:**
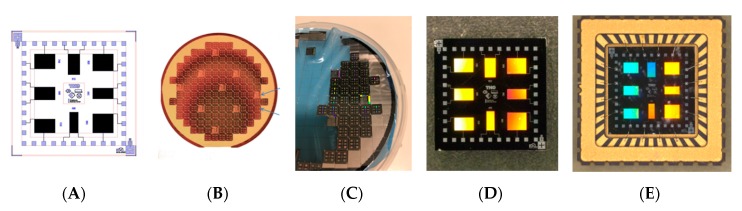
Steps in the production process of the sensor chips: (**A**) Design of the chip layout; (**B**) manufacturing of the wafer; (**C**) dicing of the wafer into individual dies; (**D**) individual die having eight interdigitated electrodes; (**E**) packaged die (= sensing chip).

The use of capacitive interdigitated electrodes (IDE) has been discussed already in several papers, ranging from gas sensors to liquid sensors [17,18,21]. This concept has the ability for miniaturization, since the electrodes can be made using complementary metal oxide semiconductor (CMOS) compatible technologies, and the read-out electronics only require a small Printed Circuit Board (PCB). So, the approach disclosed in the current paper is an important step in miniaturization of a gas sensor array. The interdigitated electrodes are manufactured on a 10 inch silicon wafer, using a standard CMOS process. First, a layer of 5 µm silicon dioxide was grown on the substrate using Plasma Enhanced Chemical Vapor Deposition. This type of silicon dioxide has a much lower porosity than thermally grown silicon dioxide, which is beneficial to reduce the absorption of water. A 1 µm thick layer of aluminum was evaporated on the silicon dioxide layer, and etched using a conventional photolithographic process. The mask that was used for exposure contained the structure of the interdigitated electrodes. The width of the conductive tracks was 1 µm, and the distance between the tracks 1 µm as well. After manufacturing, the wafer was diced in individual chips of 6 × 6 mm^2^. Two types of sensing chips were manufactured: one with eight different interdigitated electrodes, and one with four electrodes. Each of the electrode areas was different (ranging from 400 × 1000 µm^2^ to 900 × 1000 µm^2^). This generated dies having an uncoated capacitance between 3 and 7 pF. After dicing of the dies, they were packaged in a LCC04023 package from Spectrum Semiconductor Materials. The steps in the manufacturing process are shown in Figure 1.

### 2.2. Gas Absorbing Coatings

The sensing functionality of the gas sensor array depends heavily on the absorption of the target gasses in the coatings that are applied to the interdigitated electrodes. The coatings must be designed and manufactured in such a way that they absorb specific gasses and give rise to a change in dielectric constant of the material. It is known that porous materials can absorb significant amounts of gasses. This absorption is influenced by the chemical and physical interactions between the gas and surface of the porous material and depends heavily on the pore sizes and surface chemistry. These interactions can be tuned by modification of the porous structure of the materials. Coatings made from these materials are used to selectively absorb specific gasses. 

The materials that were used in the manufacturing of the coating formulations were: Polytetrafluoroethylene (PTFE) AF1600 (PTFE AF, Merck), Polymer of Intrinsic Microporosity (PIM-1, in house synthesis according to ref [22]), Zeolites H-ZSM5_26, Na-ZSM5_38, NH_4_CZP200 and NH_4_CZP800. (ACS Materials, and in-house synthesis according to ref [23]), Metal Organic Framework (MOF) ZIF 8 (in house synthesis according to ref [24]), and a fluorinated polyimide (PIFB, made according to ref [25,26]). 

Coating formulations were manufactured by dissolving the polymers in suitable solvents, such as toluene, N-methylpyrrolidon, chloroform, perfluoro compound (PFC), and dichloroethane. Some of these polymers were used as a sensing coating as such, some polymers were mixed with the additives. Weight fractions of additives in the polymer matrix ranged from 10 to 50 wt%. 

**Figure 2:**
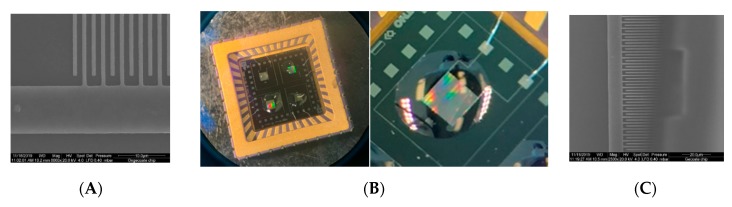
Images of an interdigitated electrode: (**A**) SEM image of empty electrode; (**B**) optical image of a (BioSpot) coated chip, before drying; (**C**) SEM image of a coated electrode (part is burned off by SEM electron beam).

The coatings were applied to the electrodes by means of several deposition methods: starting from drop-casting (~1 µL droplets), inkjet printing (Dimatix, ~50 pL droplets), and spotting (Biospot BT600, BioFluidix, ~10 nL droplets). The benefit of the spotting technique is the size of the nozzle (200 µm) and the droplets. When drop-casting the droplet size may be too big to accurately coat a single 500 × 1000 µm^2^ electrode, but some coating formulations are not suitable (yet) for spotting. The sensors presented in this paper were manufactured by drop-casting or spotting. The result of the deposition process is shown in Figure 2. The coatings that were applied to the interdigitated electrodes are listed in Table 1. 

### 2.3. Sensor Array

**Figure 3:**
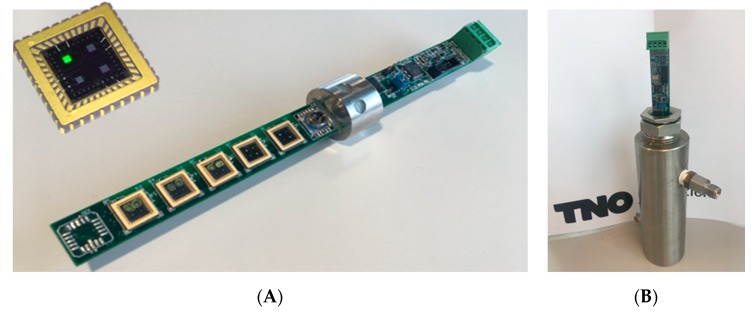
(**A**) Sensor array Printed Circuit Board (PCB) including five coated sensing chips, having ten functional electrodes, and five AD7746 chips. (**B**) Sensor array in gas cell for exposure to gas mixtures.

The sensor array was manufactured from the coated, packaged chips, and electronics that were developed by Venne Electronics (Maastricht, Netherlands) [27]. The capacitance of two of the electrodes on the chip was measured by an AD7746 Capacitance-to-Digital converter chip (Analog Devices, Norwood, MA, USA). A microprocessor was applied to the PCB to read the digital signals from the AD chips, preprocess into a table with data and transmit the data via a USB connection to an external PC or laptop. The AD chip output is a value of the capacitance relative to a reference value. The maximum absolute capacitance that can be measured using this AD chip is 21 pF. The full capacitance range is ±4 pF, with a 24-bit resolution. This relates to a capacitance resolution of 40 aF. The combination of the size of the electrode area, the dielectric constant of the coating, and the thickness of the coating must lead to an absolute capacity range between ~3 and 21 pF, in order to be suitable for the AD7746 chip. Since each AD7746 chip can interrogate two capacitive electrodes, for an array of ten electrodes, five AD chips are placed on the PCB (Figure 3A).

In addition to the values of the capacitances, a temperature and pressure reading is included in the data file. The temperature chip has a resolution of 0.1 °C, and the pressure sensor of 1 mbar. The microprocessor can send the data to the external computer with a frequency between of ca 0.5 Hz. 

The application of the sensor array will be in the natural gas grid and the fuel supply lines of an LNG engine. This means that both the temperature and pressure can fluctuate significantly. Temperature variations between −10 and 40 °C can be expected, and pressures can increase up to 8 bar(a) (= absolute pressure). The sensor array must be able to read and withstand these fluctuations. For pressure sealing, the PCB was mounted in a steel housing using an epoxy resin [28], that can withstand pressures of 10 bar(a) and 60 °C. A shield was designed to cover the exposed sensor chips, to protect them against contaminations and too high gas flows. The sensor array in a gas exposure cell is shown in Figure 3B. 

### 2.4. Exposure Experiments

The sensor array that was manufactured, having ten coated electrodes, one temperature and one pressure sensor was exposed to various gas mixtures containing methane, ethane, propane, n-butane, iso-butane, n-pentane, iso-pentane, nitrogen, and carbon dioxide. A series of 22 different gas mixtures was used to calibrate and validate the sensor array. The pressure was set at 4 bar(a), and the temperature was not regulated, but changed during the experiment (~26–30 °C) as a result of fluctuating laboratory temperature and heating of the sensor electronics. The composition of the gas mixtures was regulated by flow controllers (Bronkhorst High Tech, Ruurlo, Netherlands) and validated by the use of a gas chromatograph (Compact GC4.0, Global Analyzer Solutions, Breda, Netherlands). The sensor array was first calibrated using a selection of the gas mixtures, and subsequently validated using another set of mixtures. The calibration gas mixture is shown in Table 2.

### 2.5. Data Processing

The correlation between the sensing chip response and the gas composition is calculated according a linear relationship:

(1) $$\begin{array}{cc} Chip\;response & =Ch\\ & =\;\alpha_{1}CH_{4}+\alpha_{2}C_{2}H_{6}+\alpha_{3}C_{3}H_{8}+\alpha_{4n}nC_{4}H_{10}+\alpha_{4i}iC_{4}H_{10}\\ & +\;\alpha_{5n}nC_{5}H_{12}+\alpha_{5i}iC_{5}H_{12}+\beta +\tau T \end{array}$$

The parameters *α* are the linear correlation coefficients between the partial pressure of the gas in the mixture and the change in capacitance of the chip (in pF/mbar), and the parameter *β* is the offset value of the sensing chip, *τ* is the temperature dependency parameter of the chip, *T* is the temperature. The gas concentrations are given in mbar. All sensing chips were simultaneously used for the correlation matrix. This correlation matrix was then used to recalculate the gas concentrations in the mixtures. The multivariate linear regression approach for the simultaneous processing of all chips is as follows [29,30,31]. The matrix notation for all eight chips responses becomes;

(2) $$\left[\begin{array}{c} Ch_{1}\\ \vdots \\ Ch_{8} \end{array}\right]=\left[\begin{array}{c} \alpha_{1}^{1}\\ \vdots \\ \alpha_{1}^{8} \end{array}\;\;\;\begin{array}{c} \cdots \\ \ddots \\ \cdots \end{array}\;\;\;\begin{array}{c} \alpha_{5}^{1}\\ \vdots \\ \alpha_{1}^{8} \end{array}\;\;\;\begin{array}{c} \tau^{1}\\ \vdots \\ \tau^{8} \end{array}\right]\left[\begin{array}{c} \begin{array}{c} CH_{4}\\ \vdots \end{array}\\ \begin{array}{c} iC_{5}H_{10}\\ T \end{array} \end{array}\right]$$

*Ch*_1_ is the response of the first chip, *α*_1_^1^ the correlation parameter for the first gas and the first chip, *τ*^1^ the linear temperature correlation parameter for the first chip, and *CH*_4_ the partial pressure of methane. For calibration, many gas mixtures are required. When *n* mixtures are used for calibration, the full matrix can be written as;

(3) $$\left[\begin{array}{ccc} Ch_{1}^{1} & \cdots & Ch_{1}^{n}\\ \vdots & \ddots & \vdots \\ Ch_{8}^{1} & \cdots & Ch_{8}^{n} \end{array}\right]=\left[\begin{array}{c} \alpha_{1}^{1}\\ \vdots \\ \alpha_{1}^{8} \end{array}\;\;\;\begin{array}{c} \cdots \\ \ddots \\ \cdots \end{array}\;\;\;\begin{array}{c} \alpha_{5}^{1}\\ \vdots \\ \alpha_{1}^{8} \end{array}\;\;\;\begin{array}{c} \tau^{1}\\ \vdots \\ \tau^{8} \end{array}\right]\left[\begin{array}{cc} \begin{array}{c} \begin{array}{c} CH_{4}^{1}\\ \vdots \end{array}\\ \begin{array}{c} iC_{5}H_{10}^{1}\\ T^{1} \end{array} \end{array} & \begin{array}{cc} \begin{array}{c} \begin{array}{c} \cdots \\ \ddots \end{array}\\ \begin{array}{c} \ldots \\ \ldots \end{array} \end{array} & \begin{array}{c} \begin{array}{c} CH_{4}^{n}\\ \vdots \end{array}\\ \begin{array}{c} iC_{5}H_{10}^{n}\\ T^{n} \end{array} \end{array} \end{array} \end{array}\right]$$

*Ch*_1_*^n^* is the response of the first chip to the *n*-th gas mixture. This can be simplified to:

(4) [Ch]=[M][C]

[*M*] is the response matrix for all chips. For the calculation of the gas concentrations from the chip responses, the relation has to be rewritten into:

(5) $$\left[C\right]={\left[{\left[M\right]}^{T}\left[M\right]\right]}^{-1}{\left[M\right]}^{T}\left[Ch\right]$$

So, after obtaining the calibration matrix [*M*] from the calibration experiments, Equation (5) can be used to calculate the gas concentrations from the chip responses and temperature. The accuracy of this calculation is assessed by comparing the calculated gas concentration to the set-point gas concentration and calculate the average error. 

## 3. Tunable Filter Infrared Sensor

Tunable optical filters are microelectromechanical systems (MEMS) which enable construction of miniaturized spectrometers operating in different wavelength ranges of interest [32,33]. An example of such system is a tunable Fabry-Perot interferometer (FPI) that is suited for operation in the near infrared (NIR) spectral range [19,20]. These sensors are fiber-coupled and compact devices which can cover sufficient spectral ranges for spectroscopic analysis. With no moving parts they are also robust enough for field use. In the NIR spectral range, many molecules have characteristic spectral features and their quantitative characterization is possible. This makes real time continuous composition analysis of hydrocarbons in typical natural gas mixtures a potential application for this technique. A tunable filter IR (TFIR) sensor is realized when this kind of MEMS FPI is coupled with a continuous NIR broadband light source and a gas cell with fixed absorption length. With specific spectral analysis of recorded spectra and calibration of the system with reference gas mixtures with known concentrations sufficient selectivity is achieved in order to detect composition of a gas mixture containing hydrocarbons typically present in natural gas mixtures.

A NIRONE NIR Spectral Sensor by Spectral Engines was applied for the TFIR measurement. The sensor is operating at wavelength range 2.0–2.45 µm and it is powered by a USB source in connection with a NIR broadband light source. The device was coupled in a two pass gas absorption cell with a total absorption length of 400 mm. Gas cell temperature was stabilized to 45 °C in order to prevent condensation of longer chain hydrocarbons to cell surfaces and to stabilize the system operation for different ambient and sample gas temperatures. Gas to be measured is flowing continuously through the sample cell with volume of approx. 200 mL and the output of the gas cell is in ambient pressure. A typical gas flow is 1–2 L/min yielding a gas exchange time of approx. 10 s for the cell. Absorption spectra are recorded and the system is controlled with the Spectral Engines Sensor Control software. Time for one spectrum scan is typically 20 ms and with adopted measurement time of 2 s a total of 100 spectra is recorded and averaged. For spectral analysis, a specially constructed analysis software was implemented with a specific model for natural gas analysis. The measurement is calibrated for the following molecules and measurement concentration ranges: methane CH_4_ 0–100 vol.%, ethane C_2_H_6_ 0–30 vol.%, propane C_3_H_8_ 0–10 vol.%, n-butane n-C_4_H_10_ 0–3 vol.%, iso-butane i-C_4_H_10_ 0–3 vol.%, n-pentane n-C_5_H_12_ 0–0.6 vol.%, iso-pentane i-C_5_H_12_ 0–0.6 vol.%, and n-hexane n-C_6_H_14_ 0–0.5 vol.%.

## 4. Results

The two analytical instruments have been compared with respect to the sensitivity and selectivity for the concentrations of hydrocarbons in several gas mixtures of methane, ethane, propane, n-butane, iso-butane, n-pentane, iso-pentane, and n-hexane. 

### 4.1. Capacitive Gas Sensor Array: Calibration

The performance of the capacitive sensor was tested using a relative constant pressure of 4 bar(a) and a temperature between 26–30 °C. Both pressure and temperature are taken into account when processing the data, so small changes in these parameters do not significantly influence the outcome of the testing. In future tests a wider range of pressures and temperatures will be assessed. The functionality of the sensor array depends heavily on the chemical interactions between gas molecules and coatings. In an ideal case, the use of Henry’s law to correlate gas concentrations to the absorption of the gas in the coating (*C_c_ = H x c_m_*, in which *C_c_* is the concentration of gas in the coating, *H* the Henry constant, and *c_m_* the concentration of the gas in the gas mixture). However, in real absorption in a polymer and adsorption in porous materials the correlation is not ideal. First, the partial pressures must be used instead of the concentrations, and these are heavily influenced by the non-deal compressibility of especially the higher hydrocarbons. A detailed Equation-of-State calculation should be done to obtain an exact relation between concentration and partial pressure. In view of the expected and required accuracy of the sensor array, a simpler correlation can be used:

(6) $$p_{i}=\frac{c_{i}Z_{i}}{\sum^{\;}c_{i}Z_{i}}$$

where *p_i_* is the partial pressure of gas *i*, *Z_i_* the compressibility of the gas, and *P* the total pressure. The compressibilities for the hydrocarbons can be found in handbooks, or calculated using commercial software such as RefProp (Nist) or PVTsim (Calcep).

**Figure 4:**
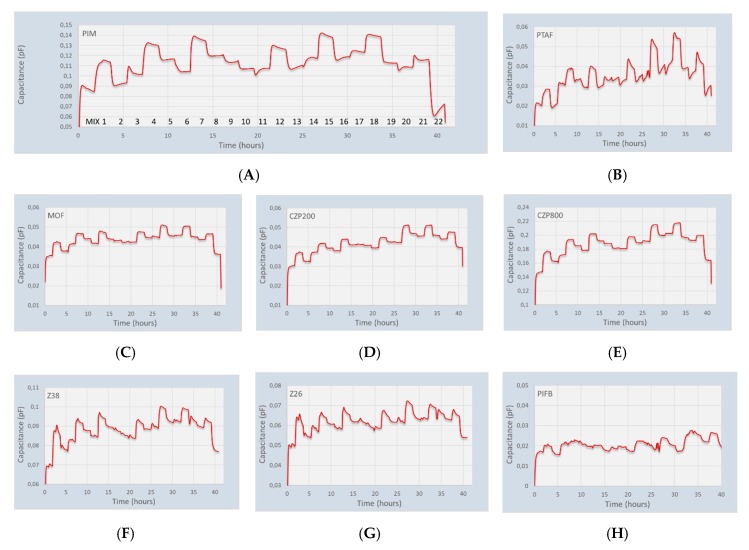
Traces of eight sensing chips when exposed to 22 different gas mixtures at 4 bar(a). (**A**) Polymer of intrinsic microporosity chip; (**B**) Fluorinated polymer chip; (**C**) Metal organic framework ZIF8 chip; (**D**) Zeolite CZP200 chip; (**E**) Zeolite CZP800 chip; (**F**) Zeolite Z38 chip; (**G**) Zeolite Z26 chip; (**H**) Fluorinated polyimide chip.

The second deviation from ideality is the gas adsorption itself into the coating. Especially the larger hydrocarbons do not follow the ideal Henry’s law, but a more real adsorption isotherm. A relatively simple adsorption isotherm is the Langmuir isotherm:

(7) $$C_{i}=C_{M}\frac{k_{i}p_{i}}{1+k_{i}p_{i}}$$

where *C_M_* is the maximum concentration of the gas that can be absorbed in the coating, *k_i_* the Langmuir constant for the gas/coating combination, and *C_i_* the gas concentration in the coating. The challenge in using the full approach (non-ideal compressibility and Langmuir adsorption) is the fact that we introduce several additional parameters that depend on pressure, temperature and composition. So, for the first assessment of the correlation between sensing chip response and composition, a linear correlation is used. The 22 gas mixtures that are listed in Table 2 are made within a gas mixing system and fed over the gas sensor array. The change in measured capacitances of eight coated sensing chips is shown in Figure 4. Most of the coated sensing chips follow the change in composition very nicely. A non-coated reference chip showed some scatter in the measurement that is not related to any gas concentration. Furthermore, some of the responses of the sensing chips changed very rapidly, but did not completely level off. The slow adsorption of the higher alkanes may be the cause of this, since it is known that smaller alkanes adsorb significantly faster into porous materials or polymers than the higher alkanes like pentane. 

**Figure 5:**
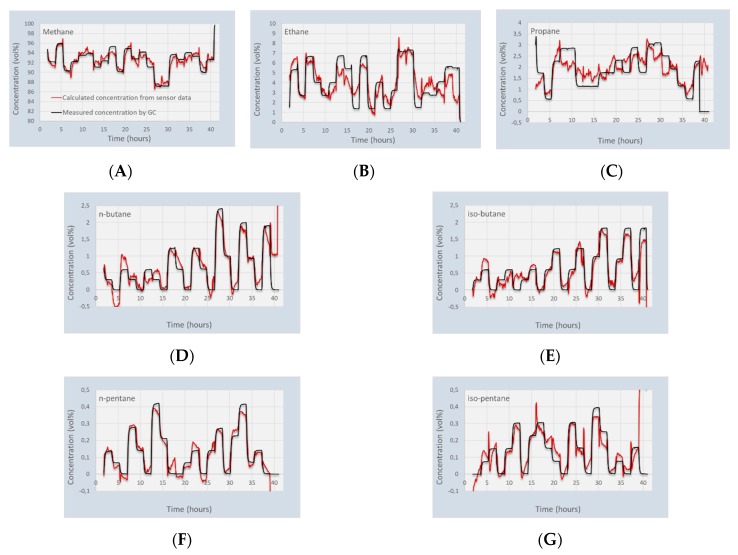
Recalculated gas concentrations in the 22 gas mixtures, compared to the values from the GC. (**A**) Methane; (**B**) Ethane; (**C**) Propane; (**D**) n-butane; (**E**) iso-butane; (**F**) n-pentane; (**G**) iso-pentane.

The data is processed according to the matrix calculations of Section 2.5. First the calibration matrix [*M*] is generated, and this matrix is used to recalculate the gas concentrations that were used for calibration. The results of this calculation is shown in Figure 5. The results from Figure 5 show that there is a clear correlation between the gas concentrations in a 7-gas mixture and the response of the sensing chips. The calculated gas concentrations correspond very well with methane and the higher hydrocarbons. However, the calculated concentrations of ethane and especially propane deviate more from the GC values. Furthermore, it appears that the responses of ethane and propane are cross-sensitive. Positive deviations of ethane correspond to negative deviations of propane, and vice versa. For the calculations of fuel quality, such as calorific value, Wobbe Index, or methane number, this may not be too detrimental. However, it may be required to add an additional sensing chip to increase the reliability of the ethane and propane detection.

The average error between the measured concentrations (using the GC data) and the calculated concentrations are listed in Table 3. The calibration experiments have shown that especially the higher hydrocarbons can be very well detected by the capacitive sensor array. However, there is still some deviation between the calculated concentrations and the set-point concentrations. In the ideal case, the calculated concentrations should follow the set-point values perfectly. There are some reasons why this difference can be found: (1) some of the sensor coatings respond very slow to the change in gas concentration. When these sensing chip values are then used for calibration, a deviation can be expected; (2) as mentioned before, ideal gas behavior is assumed, which may not be the case; (3) although the linear temperature dependency is implemented in the calculations, there may be an unknown influence of the temperature left; (4) influence of the presence of one gas on the absorption of a second gas. It is known that smaller gasses absorb first in porous structures, but may be expelled again when larger gasses with a higher affinity start to penetrate. In order to assess the applicability of the gas sensor array in real application, including all simplifications listed above, a validation experiment is done. 

### 4.2. Capacitive Gas Sensor Array: Validation

**Figure 6:**
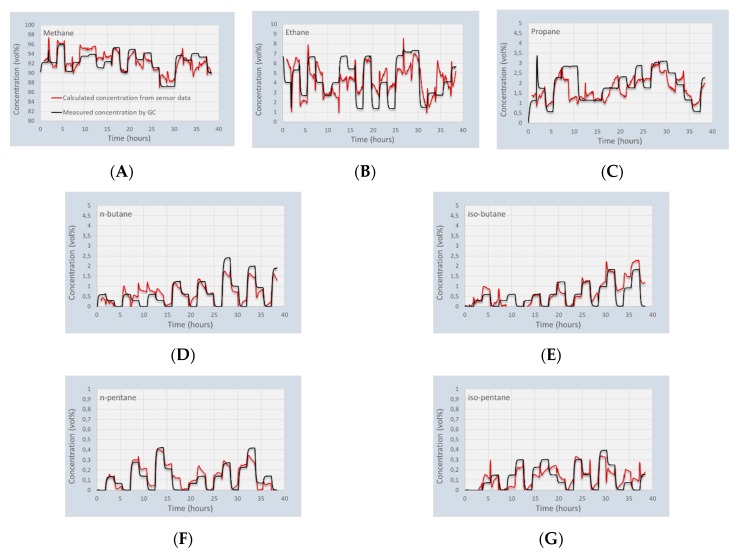
Calculated gas concentrations of the 7 gasses in the 22 gas mixtures of the validation experiment compared to GC data. The concentrations were calculated using the calibration data from Section 4.1. (**A**) Methane; (**B**) Ethane; (**C**) Propane; (**D**) n-butane; (**E**) iso-butane; (**F**) n-pentane; (**G**) iso-pentane.

The validation experiments were done using similar gas mixtures as for calibration at the same pressure of 4 bar(a) and temperature range of 25–30 °C. The processing of the data was done using the calibration matrix obtained from the calibration experiments in Section 4.1. It was seen that the response of all sensing chips is dependent on the temperature. Although this dependence is low for most chips (ca 1–2 fF/°C), the temperature difference of a few °C may disturb the sensor readings. Therefore, the raw data was corrected for the temperature differences between the calibration and validation using a linear relation, according to Equation (3). Using the thus corrected data, the concentrations of again 22 mixtures were calculated from the sensing chip data. The calculated concentration developments when the sensor is exposed to the 22 validation gasses are shown in Figure 6. In general, the correlation between the set-point and calculated gas concentrations is good, although the differences for ethane and propane are larger than in the calibration tests. It was already seen that the uncertainty in these two gasses is larger than the rest of the components in the mixtures. This behavior was not seen in measurements using only methane, ethane, propane, and n-butane [17]. Apparently, the presence of the higher hydrocarbons influences the results of these two gasses. 

For some of the gas mixtures used in the validation experiment, all calculated concentrations deviate from the set-point values, such as Mix6 at 10 h. This mix shows apparent discrepancies for methane, propane, n-butane, iso-butane, and iso-pentane. An additional assessment is needed to determine the cause of these differences: i.e., a possible mismatch between GC and exposure gasses, or an actual effect of the sensor. 

**Figure 7:**
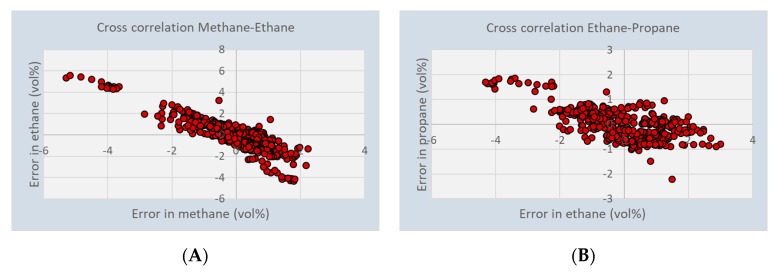
Cross sensitivity of the capacitive array sensor between (**A**) methane and ethane and (**B**) between ethane and propane.

Another reason for the large errors that are found in some parts of the experiments is the cross sensitivity of the sensor for different gasses. The significance of this is shown in Figure 7. Large negative deviations in the calculated concentration of methane corresponds to large positive deviations in the concentration of ethane. The correlation between ethane and propane shows a similar behavior. However, there appears to be hardly a dependency between the larger hydrocarbons. This indicates that the sensor array is particularly sensitive for the larger than propane hydrocarbons.

The slope in the cross correlation between methane and ethane is −1.16, and between ethane and propane −0.36. When the sensor is used for the calculation of the calorific value, and using the CVs of methane = 39.9 MJ/m^3^; ethane = 69.9 MJ/m^3^, and propane = 101.3 MJ/m^3^, the error in CV is only 0.1%, even if the uncertainty in ethane is as high as 5 vol.%. A similar behavior was found for the Wobbe Index, the effect on the Methane Number will be assessed in a forthcoming publication. 

### 4.3. Tunable Filter Infrared Sensor

**Figure 8:**
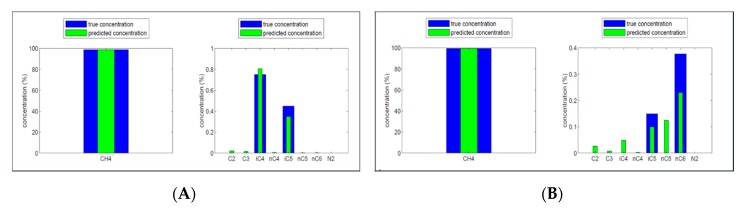
Concentration predictions for two gas mixtures containing (**A**) methane, iso-butane, and iso-pentane and (**B**) methane, iso-pentane, and n-hexane.

In order to estimate performance characteristics and especially selectivity and sensitivity of the TFIR sensor for separate gas compounds we conducted laboratory test measurements probing the systems response time, repeatability standard deviation at zero and high concentrations, lack of fit (linearity), and cross-sensitivity to other gas components. All tests are made in methane matrix. System response time for all eight studied molecules is comparable with the gas exchange time of the sample cell so well below one minute. No memory effect even for longer chain hydrocarbons with potential problems with condensation was detected. It is possible to decrease the response time with higher sample gas flow or using sample cell with smaller volume. Repeatability standard deviation (3*σ*) in zero concentration at 100 consecutive measurement point with total measurement time 200 s is below 0.02 vol.% for all compounds whereas in high concentration this parameter is below 0.1 vol.% for all compounds, with highest reading (approx. 0.09 vol.%) for n-butane. In linearity check we measure six different concentrations covering the full measurement range for all molecules. Lack of fit as a maximum difference in percentage of the full measurement concentration range is less than +/−0.6% for methane, ethane, propane and iso-butane. For n-butane the maximum difference is −1.9% of the range whereas for n-pentane, iso-pentane, and n-hexane the corresponding values are 3.6%, −3.2%, and −6.9%, respectively. Concentrations of n-pentane and n-hexane are considerably underestimated, with regression (slope) coefficients of 0.05 and 0.33, respectively, whereas for n-butane and iso-pentane the response is better, 0.80 and 0.92. For other four molecules slope is higher than 0.98. Major reason for small responses is mixing of longer straight-chain hydrocarbons in spectrum analysis and especially n-pentane and n-hexane are mostly detected as n-butane and n-butane in some level as propane. Cross-sensitivity test provides more insight in these discrepancies. In this test a total of three gas mixtures are used for all studied molecules with respect to all other molecules, considering as interfering molecules one by one. Relation of concentrations for these molecules in each case is 1:1, 3:1, and 1:3, corresponding 50%, 75% (25%), and 25 (75%) of their full measurement concentration range. For methane, ethane, propane, and iso-butane maximum response to interferent is −5% of the full range for the corresponding molecule. Ethane has this effect on propane concentration and n-butane to iso-butane concentration. For n-butane, n-pentane, iso-pentane, and n-hexane responses to interferent are larger, maximum response being −40% from n-butane to n-hexane and from propane to n-pentane. In Figure 8, spectrum analysis results are displayed for two different gas mixtures indicating this effect.

Figure 8 clearly shows that the accuracy in the detection of methane is very high, but the concentration of the other gasses contains a relevant error in the present of other disturbing gasses. This is especially the case for the higher hydrocarbons. The reason for this is the similarity of the infrared signal for the hydrocarbons with the larger chains. When the concentration of these larger gasses are also low, as is the case in natural gas or LNG, cross sensitivity becomes a significant reason for errors.

## 5. Discussion

A comparison between TFIR and Capacitive sensor has been made by the calculation of the average difference between the calculated concentrations and the set-point concentrations of the various gasses in the mixture. The results for the accuracy are listed in Table 3. 

The accuracy of the capacitive sensor array is much higher for the higher hydrocarbons than for the smaller ones, for both the calibration and the validation experiments. The average has been taken over the whole measurement time of 40 h, thus including the transitions between the gas mixtures. So, part of the large error can be explained by the longer response times of the capacitive sensor array. Especially ethane and propane have a high uncertainty related to the concentrations present. For the TFIR sensor, the accuracy is the highest for propane, but the lowest for n-butane, indicating no significant dependency of the accuracy on the molecular size. It has to be noted that the capacitive sensor array experiments were performed using 22 mixtures of 7 gasses, whereas the TFIR experiments only with mixtures of 3 gasses. In a forthcoming series of experiments both sensors will be validated against an identical series of gas mixtures containing 5–7 gasses, so the performance of both sensors can be compared better. Then, also influence of temperature and pressure will be included. 

The comparison of the presented sensors with alternative sensor solutions for the detection of the gas composition in a fuel or gas line is difficult, since there are not many examples published. Most gas composition monitoring systems are based on GC or Raman, and they perform better than the capacitive sensor with respect to selectivity (typical 0.1–0.3 vol.% accuracy), but this comes with a much higher price. Another monitoring solution comprised of various physical sensors (thermal conductivity, infrared sensor [34]) presented an accuracy of 1% in the Wobbe index. This is higher than the expected 0.1% that was presented above for the capacitive sensor. 

## 6. Conclusions

This paper describes the development and test results of a capacitive gas sensor array. The results are compared to some test results of a tunable filter infrared sensor. Although more conclusive experiments need to be done in a forthcoming paper, a few initial conclusions can be drawn. The capacitive sensor is highly sensitive for hydrocarbons larger than propane, and shows a limited cross-sensitivity between these larger hydrocarbons and methane, ethane or propane. The array of coatings that was tested shows a large cross sensitivity between ethane and methane/propane. Apparently, a coating is lacking in the array, that is especially sensitive for ethane. Nevertheless, the calculated calorific value can be calculated with a high accuracy, since errors in the ethane concentration are compensated by the methane and propane values. The TFIR sensor is more sensitive for the smaller hydrocarbons, and shows a higher cross-sensitivity for the higher hydrocarbons. It should be marked that this depends more on the chemical structure of the molecules than on the molecular size: i.e., the accuracy for the iso-alkanes is better than for the n-alkanes. This is the opposite of the accuracy behavior of the capacitive sensor.

## Figures and Tables

**Table 1:** Coating formulations that were applied to the interdigitated electrodes. PTAF = soluble polytetrafluoroethylene, PIM = polymer of intrinsic microporosity, PIFB = polyimide having fluorinated building blocks, MOF = metal organic framework.

Name	Polymer Matrix	Additive
PTAF	PTFE AF1600	–
PIM	PIM-1	–
PIFB	Fluorinated polyimide	–
MOF	PIM-1	30 wt% ZIF-8
C200	PIM-1	50 wt% NH4CZP200
C800	PIM-1	50 wt% NH4CZP800
Z26	PIM-1	50 wt% H-ZSM5_26
Z38	PIM-1	50 wt% Na-ZSM5_38

**Table 2:** Gas mixtures and concentrations (in vol.% as measured by the gas chromatograph) of the calibration experiments.

Mixture	CH_4_	C_2_H_6_	C_3_H_8_	*n*-C_4_H_10_	*i*-C_4_H_10_	*n*-C_5_H_12_	*i*-C_5_H_12_
Mix1	92.21	5.32	1.74	0.30	0.30	0.13	0.00
Mix2	95.99	2.71	0.56	0.00	0.60	0.07	0.07
Mix3	90.31	6.67	2.27	0.60	0.00	0.00	0.15
Mix4	92.25	4.03	2.84	0.30	0.30	0.28	0.00
Mix5	93.54	2.72	2.85	0.00	0.60	0.14	0.15
Mix6	93.94	4.02	1.14	0.60	0.00	0.00	0.30
Mix7	91.10	6.73	1.14	0.31	0.30	0.42	0.00
Mix8	92.40	5.42	1.14	0.00	0.60	0.21	0.23
Mix9	95.33	1.38	1.75	1.23	0.01	0.00	0.30
Mix10	90.16	6.73	1.75	0.61	0.60	0.00	0.15
Mix11	94.94	1.38	2.31	0.00	1.22	0.07	0.08
Mix12	92.79	4.06	1.76	1.24	0.01	0.14	0.00
Mix13	94.20	1.38	2.88	0.62	0.61	0.00	0.31
Mix14	93.48	3.24	1.76	0.00	1.23	0.14	0.15
Mix15	87.12	7.15	3.04	2.41	0.01	0.27	0.00
Mix16	87.21	7.31	3.10	1.00	0.99	0.00	0.39
Mix17	93.70	1.49	2.50	0.00	1.83	0.23	0.25
Mix18	92.69	2.98	1.91	1.99	0.01	0.42	0.00
Mix19	94.09	2.75	1.15	0.94	0.92	0.07	0.08
Mix20	93.34	4.11	0.58	0.00	1.83	0.14	0.00
Mix21	90.00	5.64	2.28	1.91	0.01	0.00	0.16
Mix22	92.66	5.50	0.00	0.01	1.83	0.00	0.00

**Table 3:** Accuracy of the hydrocarbon concentrations derived from the (1) experiments with the capacitive sensor array: calibration experiments in Section 4.1, and from the validation experiments in Section 4.2, using mixtures of 7 gasses; (2) the experiments with the tunable filter infrared spectrometer, using mixtures of 3 gasses. Calculated as average difference between calculated concentration and set-point, in vol.%.

Gas	Capacitive Calibration	Capacitive Validation	TFIR Validation
CH_4_	0.50	1.20	0.17
C_2_H_6_	0.78	1.30	0.16
C_3_H_8_	0.39	0.39	0.03
*n*-C_4_H_10_	0.18	0.25	0.36
*i*-C_4_H_10_	0.14	0.27	0.05
*n*-C_5_H_12_	0.03	0.04	0.10
*i*-C_5_H_12_	0.03	0.06	0.07
*n*-C_5_H_12_	–	–	0.10
